# Notes on Kangaroos

**Published:** 1894-03

**Authors:** A. W. Whitehouse


### NOTES ON KANGAROOS.

By A. W. Whitehouse.*

The case of Jack, the boxing kangaroo, who died at the Musée, in the autumn, of a general inflammation of the respiratory tract, came into my hands for a partial autopsy, and in dissecting his trunk I found so much that was interesting and unique, that I have embodied it in a communication.

Before giving a history of the case, I will review a few facts about the habits of the kangaroo in his normal state. The Australian climate is semi-tropical and dry; the kangaroo is purely herbivorous, and the seed being scanty he has to cover large distances in finding it; he not only grazes, but grubs up roots with his claws; he will nibble bark, and, squirrel-like, will hold fruit or

---

* Read before the Ontario Veterinary Medical Society.

corn-cobs between his fore-paws. The books differ as to whether he chews the cud or not; but according to Mr. Tait (Jack's proprietor) he certainly does; and the conformation of the stomach puts the question, I think, beyond a doubt. You will remember, then, that his natural climate is hot and dry; his natural food bulky and innutritious.

I will first give you all that I learnt from Mr. Tait as to his early history and management; and the course of his fatal illness, and the treatment employed—very kindly furnished by Dr. Cooper of North Toronto. Mr. Tait caught him himself about 200 miles from Sydney, N. S. W., when a "joey" or young fawn, and trained him to spar. At the time of his death he was about 3½ years old.

He used to feed him regularly on oats, hay, green corn, carrots and turnip tops; and considering that he needed extra nourishment, on account of his exertions, added a daily ration of three quarts of milk.

To fill an engagement with him at Moore's Musée, he left Chicago on Saturday, October 21st, rested Sunday the 22nd at Detroit, and started the same evening for Toronto, Jack being at that time apparently in perfect health. Jack used to travel in a large deal box, about a 7 foot cube. After seeing him safely stowed in the baggage car of the first section, Mr. Tait and his partner, by some mistake, found themselves in the second section of the train; and unfortunately arrived in Toronto early on Monday, October 23rd, about three hours later than their charge. The weather was very cold and raw. They found that he had been delivered at the Musée, but, no one having any instructions, his box had been merely left in the open yard. A further delay ensued, owing to the difficulty of getting the box upstairs. Removal to a shed only aggravated matters, as there was a strong draught; so it resulted that the kangaroo was exposed to bitter cold for about five hours, with only the protection of a comparatively thin deal box—cramped up and unable to warm himself by exercise. Almost immediately after this the kangaroo was noticed to be unwell. In his performance that afternoon he frothed considerably at the mouth, and this symptom, together with more distress than usual, was noticed in the evening. He seemed to have some difficulty in swallowing, and ate very little. Mr. Tait supposed he might have something wedged between the molars, or have eaten some substance irritating to the mouth. So the next morning—Oct. 24th—he called in Dr. Cooper, of Yorkville Avenue.

The résumé of the case, as given me by Dr. Cooper, is as follows:

October 24.—First saw Jack at 12 noon. Examined mouth, by Mr. Tait's request; found neither foreign body lodged nor signs of local irritation, but a considerable flow of saliva. Temperature by the mouth 103 degrees. Administered 10 gr. quinine in a pint of milk, given by means of a syringe. Diagnosed respiratory trouble of some kind. From this time on the animal was completely off his feed, taking food in his mouth only to munch it and put it out; receiving only artificial nourishment.

Present at afternoon performance. Patient dull at first; when he did spar, quickly distressed, with labored breathing; wheezed and snuffled, but did not cough then or at any period of the, disease. (This is accounted for by an examination of the larynx which shows the total absence of vocal chords.) Strongly recommended Mr. Tait to discontinue the performance. He felt this hardly fair to the public, after the extensive advertising.

4.30 P. M. Administered 10 gr. quinine in milk as before. 10 P. M. Witnessed evening performance. Jack would not box at all, and his breathing was very labored. This was his last performance.

Oct. 25.—9.30 A. M. Temperature 103½. Added 10 gr. Iodide of Potassium to morning and evening dose, giving the 10 gr. quinine morning, noon and night. Continued this treatment to the end.

Oct. 26.—Patient having eaten nothing, added to each dose 3 ounces of arrowroot, making 9 ounces a day, and continued this treatment to the end. During the course of the disease the bowels acted with normal regularity. For the first two or three days the fæces were covered with mucus; latterly they were normal. The urine was passed regularly. The temperature rose steadily but slowly to 104½.

On Friday the 27th and Saturday the 28th a rusty discharge, mixed with blood, issued from the nostrils, but the patient seemed better in himself, hopping around, snuffing at his food, but still unable to swallow solids. Early on Sunday the 29th there was collapse, and every sign of approaching death, which took place at 10.30 A. M.

On Monday morning I heard of Jack's death, and was fortunate enough to be present at the post-mortem, which was performed at a taxidermist's by Dr. Cooper, in the presence of Dr. Sweetapple.

The cause of death was clearly enough shown by the post-mortem appearances; but if it had not been for an engagement which compelled Mr. Tait to leave early in the afternoon, a neater and more satisfactory job could have been made. Mr. Tait naturally wished to see the post-mortem, and naturally, also, he wanted the skin preserved; if there had been time enough the taxidermist would have made a small incision in the skin of the belly, disarticulated the limbs and neck, and turned the trunk over, intact. As it was, Dr. Cooper had to conduct the post-mortem solely through the longitudinal incision (about seven inches in length) in the abdomen, breaking down the diaphragm to reach the lungs.

Of course, in speaking of the viscera of an animal so little known, it is hard to say what is normal and what is not, but the point mainly noticeable in the abdominal cavity was the condition of the small intestine. The duodenum was a most brilliant yellow, being infiltrated with bile; the jejunum was only a little less so, while in the ileum the color gradually shaded off into normal. This condition was not noticeable in the stomachs. The liver was remarkably full of bile, which exuded in considerable quantities on pressure. The other abdominal viscera, with the possible exception of the spleen, which was soft and pulpy, were apparently normal. There was a little of the usual blood-stained serum in the abdominal cavity. I afterwards noticed two ulcerated patches, one about the size of a quarter of a dollar and the other a little smaller, in the true stomach, close to the pyloric orifice; they had rather the appearance of typhoid ulcers; were slightly indurated, and there was no inflammatory ring round them. To these I attach no importance.

On breaking through the diaphragm there was at once noticed in the pleural cavity a large quantity of fluid, perfectly opaque, and of the rich crushed strawberry tinge described as characteristic of contagious pleuro-pneumonia in cattle. The pleura were dark and mottled with purple-black patches; numerous adhesions were found from the costal and diaphragmatic pleura to those of the lungs. These organs were in a state of red hepatization, though not equally so all over; no part was healthy, but patches could easily be distinguished. The mucous membrane lining the trachea and larynx were black—no other word properly describes it; no swelling or œdema was noticeable. The whole body was remarkably well nourished, and while the fat around the viscera was not abnormal in quantity, it led us to remark that he could have stood starvation well for another week or ten days.

So far, the explanation of the case seems tolerably clear. The animal was kept in high muscular condition, which rather predisposes to inflammatory diseases, and was suddenly exposed to a severe chill—the milk that was regularly given him was not normal food; and it is just possible that milk will act on adult herbivora in a similar way that stimulants act on a human being, rendering the respiratory mucous membrane less resistant to inflammatory attacks.

The bilious condition of the intestines I regard as a symptom, but not a serious one, the liver being sympathetically irritated and there being no bulky food to carry away the secretion. Possibly even the secretion may have been merely normal, but not removed. Or the whole condition may have been a post-mortem staining or infiltration. According to old-fashioned pathology, the animal underwent quite sufficient exposure to account for the respiratory inflammation and death. Unfortunately for this explanation, Dr. Caven made examinations of the lungs, and found in lung scrapings considerable numbers of the bacillus of malignant œdema. This bacillus is very like that of anthrax, but the ends are rounded. After very careful examination of authorities, I can find no account of malignant œdema arising naturally. The bacillus and the disease were discovered by Koch, who was injecting earth into rabbits. Frequently this had no effect; in some cases they died of tetanus; in a few of malignant œdema. The course of the disease resembled that of anthrax, with death invariably in two days. The post-mortem showed enlargement of the spleen, and very rapid putrefaction. Though there is no literature on the subject of malignant œdema with the exception of these artificial cases, Mr. Babcock during the summer noticed some sudden deaths among horses, which, on investigation by Dr. Caven, proved to have been caused by this same bacillus. Mr. Babcock will, I am sure, be glad to tell you what he knows about these cases, after I have finished. The course of the disease in our kangaroo being so different from that of malignant œdema, and the meat being in such excellent condition for several weeks after death, I was very much puzzled, indeed. It transpired that the lungs lay in an ash-barrel, together with a lot of rubbish, for some hours before Dr. Caven got them, and this may have been the source of the bacillus of malignant œdema. A further microscopical examination of the lung scrapings revealed, in addition to the bacillus of malignant œdema, a thinner rod, a common putrefactive form, and, besides these, a minute diplococcus, probably identical with the coccus of human pneumonia.

After this, Dr. Caven examined an anatomical section, and pronounced the condition to be identical with acute croupous pneumonia in the human being, though he still holds that the lungs were not in a bad enough condition to kill the patient, had he been human. However, we know that it takes comparatively little to carry off a domesticated wild animal.

Having about exhausted the pathological question, I will describe some of the most interesting points in normal anatomy that I picked up at the autopsy and subsequently in dissection. The taxidermist needed the head and the limbs, in order to set up the specimen, so I only succeeded in carrying off the trunk—including, however, the tongue and pharynx (in a rather dilapidated condition). I noticed the following points about the head and limbs. The incisor teeth are six in the upper jaw with the muzzle very sharply pointed in front, and the nippers considerably the longest; in the lower jaw there are only two incisors, very long and large, and placed horizontally. It is said that the rami of the lower jaw never unite, and there is motion between them, so that when the grass is very short, the animal separates the two lower incisors, applies them to the ground, draws them together, and so nips off what he can. As I did not get the lower jaw, I cannot certify to the truth of this. It is characteristic of all marsupials to have an uneven number of incisors in the upper and lower jaws. The molars are five on each side of each jaw, and appear to be very much like those of a horse. The arm and forearm are short and very muscular, tapering to a tiny wrist, from which springs a hand with five powerful claws, all working in the same direction; in quality of skin and shape of talons much resembling the foot of an eagle or hawk. The hind foot is curious. From the hock to the toe, the lower surface is usually in contact with the ground. At the tip of the toe is a single and very formidable-looking nail, about the size of one-half of a sheep's foot. Inside of the top of the fetlock is a dew claw, small and unimportant, like that of a dog, but double, and terminating in two sharp, sickle-shaped nails —outside, and a little lower down, there is a single similar claw.

There is a callous or pad just back of the toe, and another, which almost joins it, at the fetlock. A third callous protects the lower surface of the hock or heel. Comparing this last callous with the chestnut on the hind leg of the horse, one observes that there is very little difference in position, and it leads one to speculate whether the ergots and chestnuts may not be put down as evolutionary remains of callouses rather than rudimentary digits

The tail, as is well known, is enormous; about eight inches in diameter at the base, and over three and a half feet in length.

Dr. Caven carried off the lungs, heart, liver and spleen for microscopical examination, and I had no opportunity to look at them thoroughly. The first three showed no marked anatomical peculiarity, but the spleen is of a curious shape, being a kind of three-legged arrangement In the respiratory tract the chief peculiarity is in the larynx, which is destitute of vocal chords, rendering the animal voiceless. We were unable to find any hyoid bone, its place being apparently represented by a half ring of cartilage, lying in front of the epiglottis, and garnished with two small horns of yellow elastic cartilage. Unfortunately, these were both broken off in dissecting out. The soft palate holds an intermediate place between those of the horse and ox; it is doubtful if expiration can take place through the mouth.

In the alimentary tract the main peculiarity lies in the stomach. This has all the appearance of an intestine. It lies on the left side, running almost the whole length of the abdomen, and curved back on itself. About the anterior four-fifths must be taken as representing the false stomachs of the ox, being lined with cuticular membrane and much sacculated The true stomach is small. There is no vestige of an ileo-cæcal valve, and the cæcum is curved for its whole extent.

The pelvic cavity is naturally rather small, and is rendered still more so by cushions of fat which surround the pelvic organs, and are accurately moulded on them. This is evidently a very beautiful provision to prevent dislocation or churning of the contents during the enormous leaps and bounds of the animal. The testicles are in the usual place and in themselves present nothing remarkable ; the penis, however, which I will describe later, is internal, lying in the anus, and only being protruded when in use. Attached to the testicles is an enormous cremaster muscle, nearly an inch in breadth and about a quarter of an inch thick. As in the horse its superior attachment is to the iliac fascia. Dissecting the inguinal region, we found the inner border bounded by a bone. This bone is called the epipubic. It is a V shaped arrangement, attached to the pubis and forming the end of the common prepubian tendon. From here it branches, and strengthens the internal walls of the inguinal canal.

I said to myself, "What on earth is all this arrangement for ? Why does the animal want to withdaw his testicles, and why all this leverage arrangement ?" It turns out that though the apparatus has apparently no use in the male, it is very important in the female. The following is the explanation. The period of gestation is very short, being between 20 and 40 days; and the young of a female weighing more than 100 pounds will not be more than an inch in length. When born this little creature is fairly well developed about its anterior quarters, the hands and mouth being quite distinguishable. The hind legs and tail are represented by three little lobes. The mother picks this little thing up in her lips, holds her pouch open with her hands, and places it on a nipple, of which there are four. The little animal is not sufficiently developed to suck of itself; the mammary glands are therefore supplied with cremasters, even stronger than those of the male, and these, working over the epipubic bones as a pulley, force the milk into the young one's mouth. The young remain in the pouch for some months, and are developed there; it is more than a year before they leave it finally.

Another point of interest is to be found in the vertebræ of the tail. These show a reversion in their lower part to the complete vertebra of Owen; that is to say they possess parts representing the pleurapophysis and haemapophysis, or, to speak more simply, the ribs and sternal segments. Articulating with every two coccygeal vertebræ below, we find what is called the chevron bone, a V shaped bone, allowing between it and the lower surface of the vertebra a passage for blood-vessels.

Returning to the genito-urinary tract, the following peculiarities were noticed. The ureters open into the bladder, one just above the other, and both just above the urethra; so that these orifices form a straight line instead of the usual trigonous vesicæ. We traced the vasa deferentia up from the testicle into the lateral ligaments of the bladder, but as they were so small that it was impossible to probe them, we failed to find their point of entrance into the urethra. There were apparently no visiculæ seminales. Just posterior to the bladder two large glands opened into the urethra; these are probably Cowper's glands. The urethra then passes completely through the prostate, which is pear-shaped, and opens into the urethra by a number of small papillæ, arranged in four longitudinal rows The penis lies just within the anus, being situated analogously to the female clitoris in the vulva; it is provided above with powerful retractor muscles (thicker than a pencil) which pass on each side of the rectum and are attached to the sacrum. When protruded the penis is pointed and about seven inches long. Erection (according to Mr. Tait)

causes it to curve forwards till it is in the ordinary position seen in other animals. Inside the urethra is a process of erectile tissue, about the length and thickness of a match. The purpose of this I cannot explain.

